# Supplementary material for: PD-L1 degradation is regulated by electrostatic membrane association of its cytoplasmic domain
Source: Nat Commun. 2021 Aug 24;12:5106. doi: 10.1038/s41467-021-25416-7 (PMC8384847; doi:10.1038/s41467-021-25416-7)
Supplement: Supplementary file 1 — Supplementary Information [file 41467_2021_25416_MOESM1_ESM.pdf]

Supplementary Information

PD-L1 degradation is regulated by electrostatic membrane association of its cytoplasmic domain

Maorong Wen[1]†*, Yunlei Cao[1,2]†, Bin Wu[3]†, Taoran Xiao[1,2], Ruiyu Cao[1,2], Qian Wang[1,2], Xiwei Liu[1,2], Hongjuan Xue[3], Yang Yu[3], Jialing Lin[4,5], Chenqi Xu[1,2], Jie Xu[6], Bo OuYang[1,2]*

[1] State Key Laboratory of Molecular Biology, Shanghai Institute of Biochemistry and Cell Biology, Center for Excellence in Molecular Cell Science, Chinese Academy of Sciences, Shanghai 200031, China

[2] University of Chinese Academy of Sciences, Beijing 100049, China

[3] National Facility for Protein Science in Shanghai, ZhangJiang lab, Shanghai Advanced Research Institute, Chinese Academy of Sciences, Shanghai 201203, China

[4] Department of Biochemistry and Molecular Biology, University of Oklahoma Health Sciences Center, 940 Stanton L. Young Boulevard, Oklahoma City, OK 73126, USA

[5] Stephenson Cancer Center, 800 Northeast 10th Street, Oklahoma City, OK 73104, USA

[6] Institutes of Biomedical Sciences, Fudan University, Shanghai 200433, China

† These authors contributed equally.

* Correspondence should be sent to Maorong Wen, Shanghai Institute of Biochemistry and Cell Biology, Center for Excellence in Molecular Cell Science, Chinese Academy of Sciences, 320 Yueyang Road, Shanghai, China 200031, Telephone: 86-021-54920143, Email: mrwen@sibcb.ac.cn and Bo OuYang, Shanghai Institute of Biochemistry and Cell Biology, Center for Excellence in Molecular Cell Science, Chinese Academy of Sciences, 320 Yueyang Road, Shanghai, China 200031, Telephone: 86-021-54920143, Email: ouyang@sibcb.ac.cn

Table of Contents

Supplementary Figure 1……………………………………………………………..………S2
Supplementary Figure 2……………………………………………………………..………S3
Supplementary Figure 3…………………………………………………………….……S4
Supplementary Figure 4……………………………………………………………..………S5
Supplementary Figure 5…………………………………………………………….……S6
Supplementary Figure 6…………………………………………………………….……S7
Supplementary Figure 7…………………………………………………………….……S8
Supplementary Figure 8…………………………………………………………….……S9
Supplementary Table 1 ……………………………………………………….….…….. S10
Supplementary Figure 9........................…………………………………….…….…….. S12
Supplementary References……………………………………………………………S13

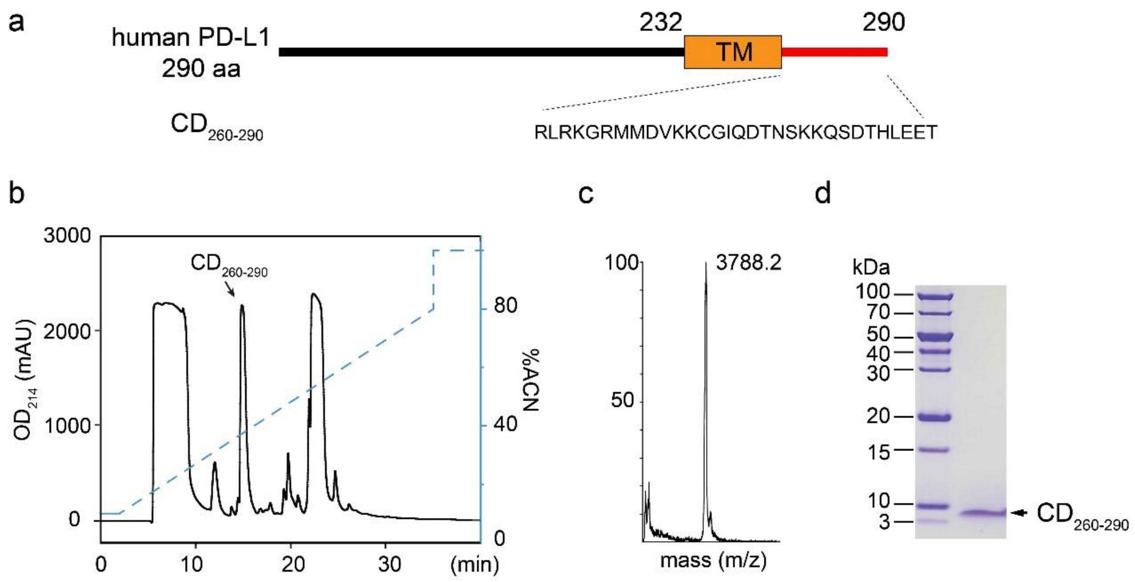

Supplementary Figure 1. Purification of $CD_{260\text{-}290}$ protein.

(a) $CD_{260\text{-}290}$ sequence (residues 260-290) used to prepare the fusion protein for NMR and biochemical experiments.

(b) HPLC profile of $CD_{260\text{-}290}$ protein eluted with the gradient of Buffer B from 10 to 80% for 33 min and 100% for 5 min, respectively. The elution time, optical density at 214 nm (OD_{214}), and % of acetonitrile (%ACN) in the elution buffer is indicated on the bottom, left and right axes, respectively. Black arrow indicates the $CD_{260\text{-}290}$ fraction. Buffer A: 10% (v/v) ACN with 0.1% (v/v) trifluoroacetic acid (TFA); Buffer B: 80% (v/v) ACN and 0.1% (v/v) TFA.

(c) Mass spectrometry analysis of HPLC purified $CD_{260\text{-}290}$ protein. The molecular weight determined for $CD_{260\text{-}290}$ is 3788.2 Da, consistent with the theoretical molecular weight of 3788.33 Da.

(d) SDS-PAGE analysis of HPLC purified $CD_{260\text{-}290}$ protein. The lyophilized $CD_{260\text{-}290}$ powder was dissolved in gel loading buffer, and incubated at 100 ^{o}C for 5 min before SDS-PAGE. The $CD_{260\text{-}290}$ band was detected by Coomassie blue staining and indicated by an arrow.

All data shown above are representative of at least three independent experiments.

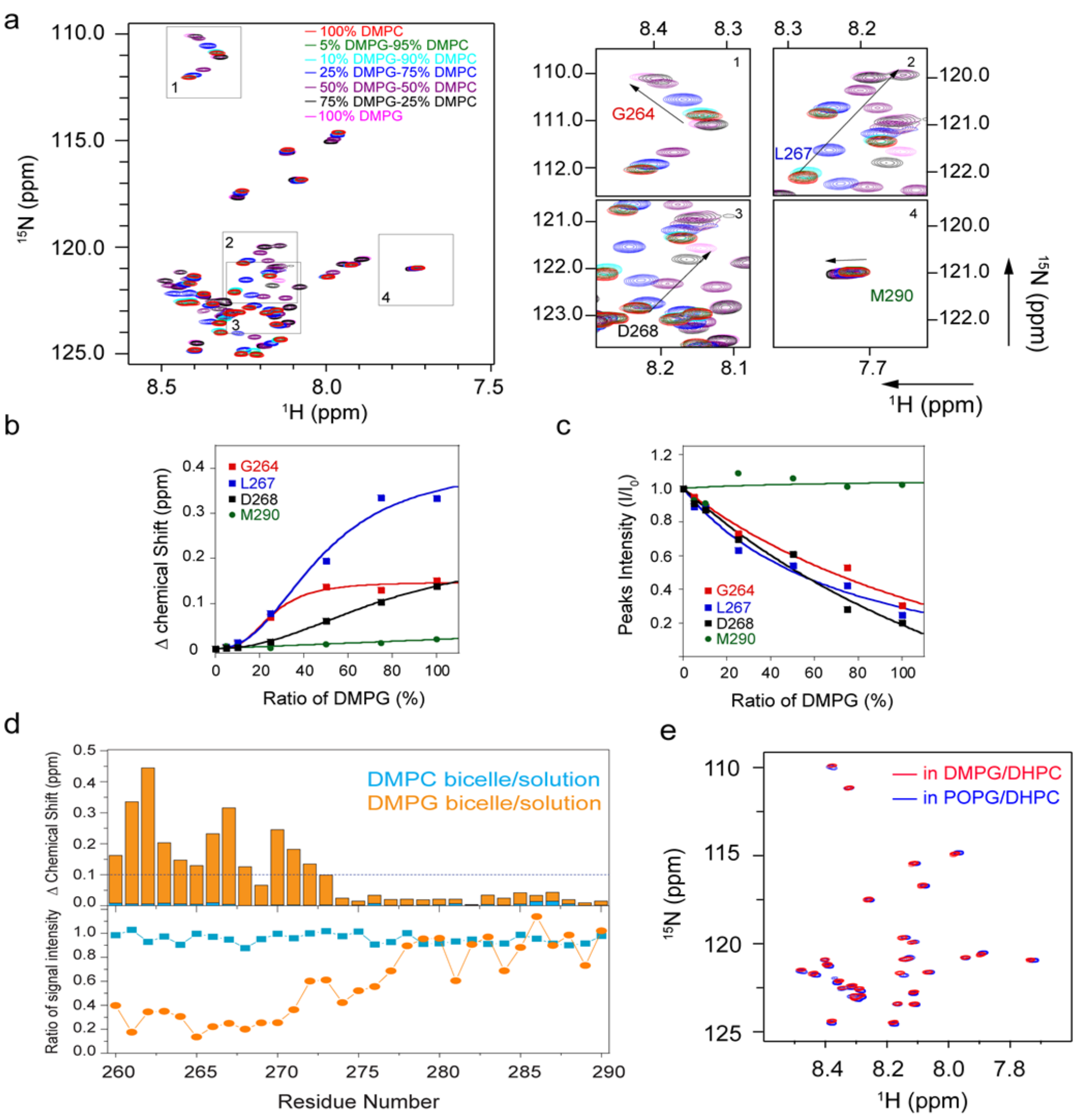

Supplementary Figure 2. Lipid composition effects on $CD_{260-290}$.

(a) Superimposed 2D ^{1}H-^{15}N TROSY-HSQC spectra of $CD_{260-290}$ in 100% DMPC/DH^{6}PC (red), 5% DMPG-95% DMPC/ DH^{6}PC (green), 10% DMPG-90% DMPC/DH^{6}PC (cyan), 25% DMPG-75% DMPC/DH^{6}PC (blue), 50% DMPG-50% DMPC/DH^{6}PC (purple), 75% DMPG-25% DMPC/DH^{6}PC (black) and 100% DMPG/DH^{6}PC (magenta) bicelles. The q values for the bicelles are all 0.8. The right panels 1-4 show the same spectral regions labeled on the full spectrum, highlighting the chemical shift changes for G264, L267, D268 and M290, respectively.

(b) Plots of chemical shift changes versus DMPG percentage for the residues labeled in (a).

(c) Plots of intensity changes versus DMPG percentage for the residues labeled in (a).

(d) Comparison of site-specific amide backbone ^{15}N NMR chemical shift changes (top) or intensity changes (bottom) between DMPC/DH^{6}PC bicelles versus in the solution (blue) and DMPG/DH^{6}PC bicelles versus in the solution (orange).

(e) The comparison of 2D ^{1}H-^{15}N TROSY-HSQC spectra of $CD_{260-290}$ in DMPG/DH^{6}PC (q=0.8) bicelles (red) and POPG/DH^{6}PC (q=0.8) bicelles (blue).

Source data are provided as a Source Data file.

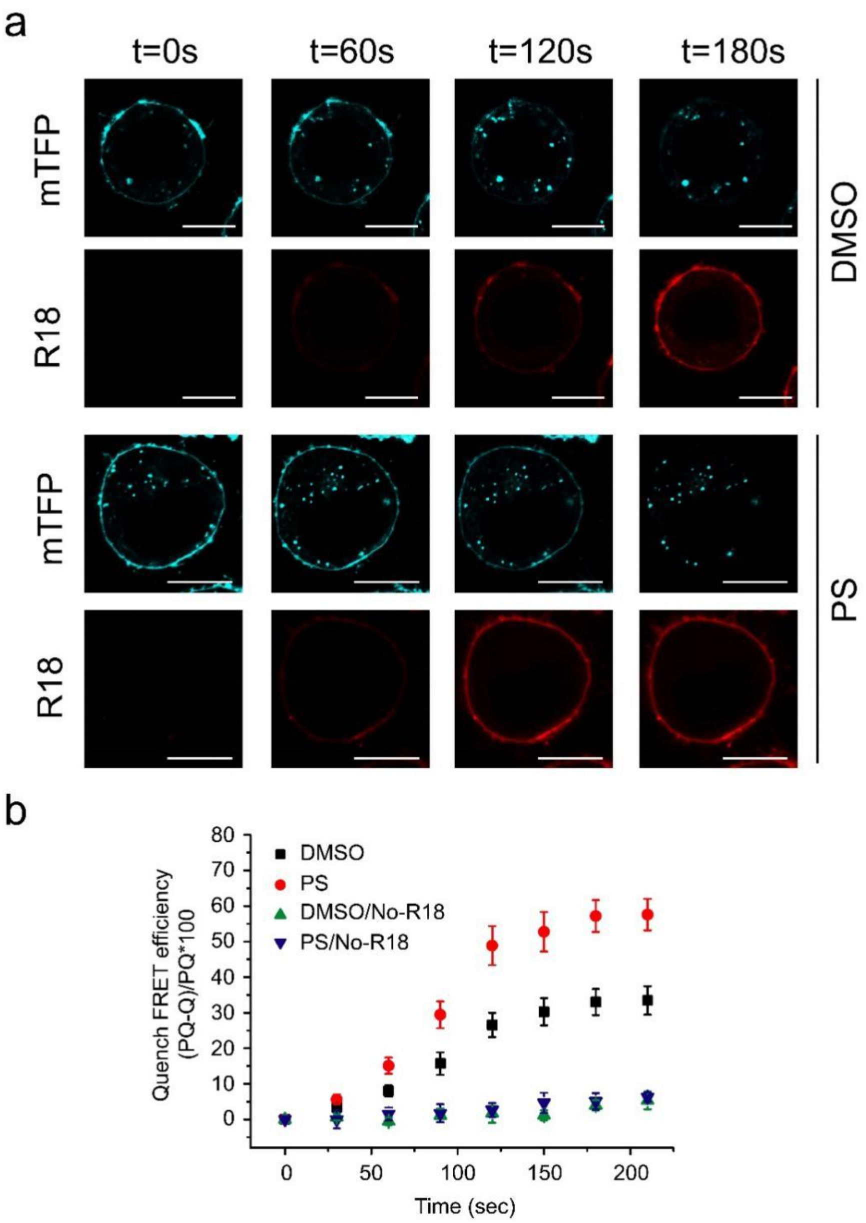

Supplementary Figure 3. FRET approach to examine PD-L1-CD and membrane interaction when cell were treated by PS.

(a) Measurement of FRET efficiency with the donor quenching approach. Cells were adhered to glass coverslips. R18 was then injected and images taken at 30 s intervals for both mTFP and R18 channels. FRET efficiency was calculated based on the reduction of mTFP fluorescence due to energy transfer to the R18 acceptor. Scale bars, 5 μm.

(b) FRET efficiencies were measured at 30 s intervals after R18 was injected. 5 cells for DMSO/No-R18, 6 cells for PS/No-R18, 11 cells for DMSO control and 13 cells treated with 5 μM PS for 2 h were counted. Data are represented as the mean ± SD.

Source data are provided as a Source Data file.

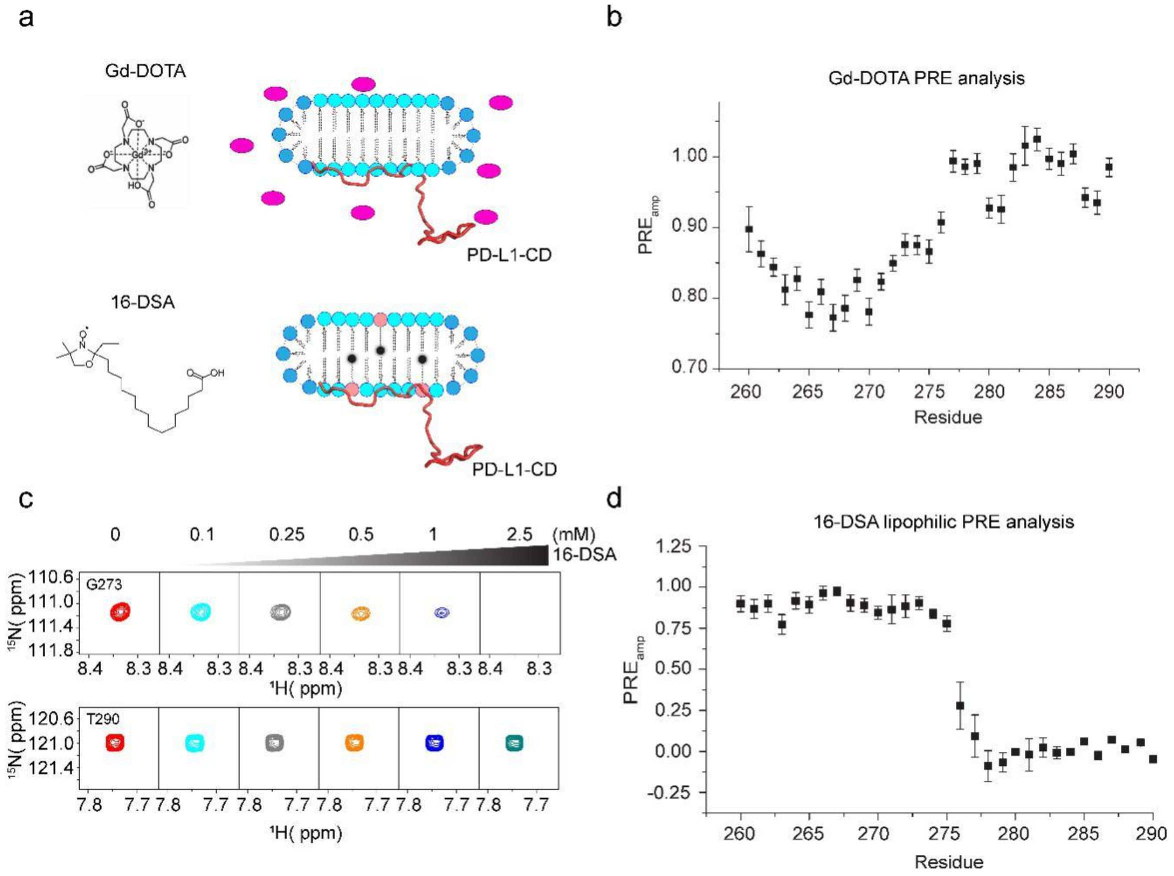

Supplementary Figure 4. PRE analysis of $CD_{260-290}$ partition into lipid bicelles.

(a) Schematic illustration of titrating the bicelle-bound $CD_{260-290}$ with the water-soluble Gd-DOTA (magenta ovals) or the lipophilic 16-DSA PRE probe (pink balls). The black dot represents the nitroxide group in 16-DSA.

(b) PRE_{amp} versus residue number plot obtained from the Gd-DOTA titration. The intensity vs [Gd-DOTA] data were fitted by the exponential decay function (Equation 1) to determine the PRE_{amp}. The results are represented as the PREamp values ± standard error.

(c) Parts of 2D ^{1}H-^{15}N TROSY-HSQC spectra of $CD_{260-290}$ in DMPG/DH^{6}PC bicelles measured at the indicated 16-DSA concentrations at ^{1}H frequency of 700 MHz NMR spectrometer at 30°C. The data show that the addition of 16-DSA causes significant decreases of the spectral peak intensities for G273 near the middle of $CD_{260-290}$ but not for T290 at the C-terminus. The data from 3 and 5 mM 16-DSA titrations were omitted since these high concentrations of 16-DSA completely eliminated the spectral peak from G273.

(d) PRE_{amp} versus residue number plot obtained from the 16-DSA titration. The intensity vs [16-DSA] data were fitted by the exponential decay function (Equation 1) to determine the PRE_{amp}. The results are represented as the PREamp values ± standard error.

Source data are provided as a Source Data file.

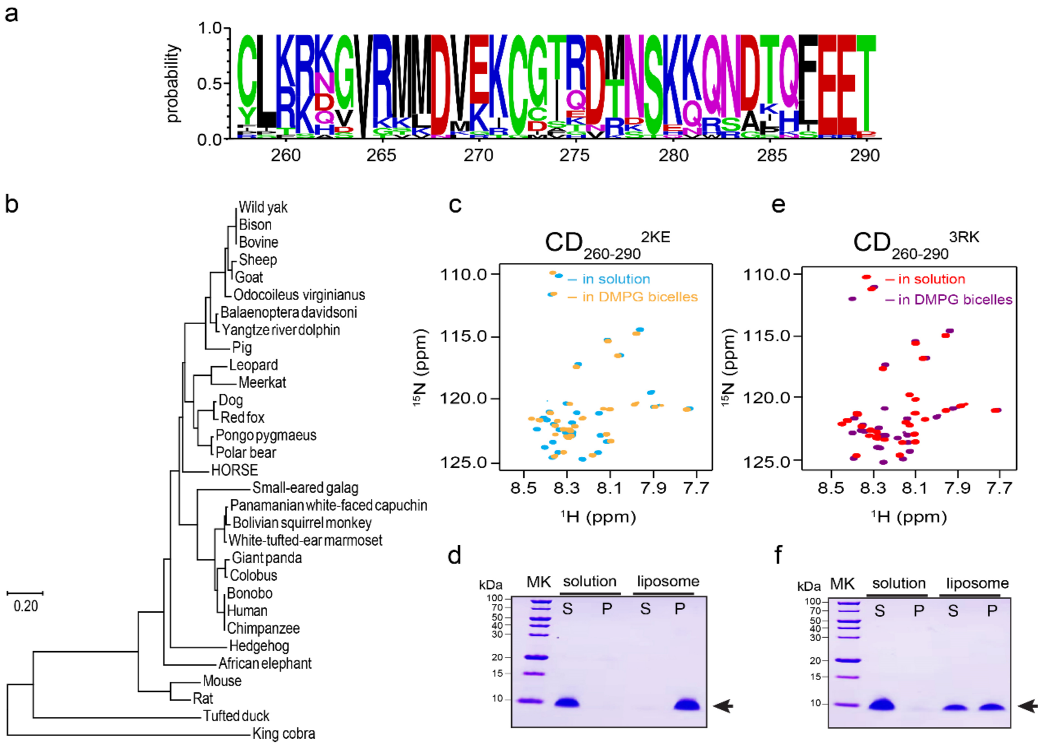

Supplementary Figure 5. PD-L1-CD sequence conservation and effect of additional mutations on the membrane interaction; related to Figure 3.

(a) Sequence logos illustrate the degree of amino acid conservation among PD-L1-CD sequences from 31 species[1]. The height of each letter stack indicates the relative conservation of that amino acid residues at that position. Different amino acid side-chain categories are color coded with basic (blue), acidic (red), hydroxyl/thiol-containing (yellow), carboxamide (purple), small (green), and aromatic/hydrophobic (black).

(b) Phylogenetic relationship among 31 PD-L1 homologs. The tree is constructed based on the alignment of PD-L1 and generated by MEGA7[2]. The branch lengths are roughly proportional to the amount of sequence difference, as calculated using the program Bioedit and MEGA7 with full-length sequences.

(c) Superimposed 2D ^{1}H-^{15}N TROSY-HSQC spectra of $CD_{260\text{-}290}^{2KE}$ in solution (blue) and in DMPG/DH^{6}PC bicelles (q=0.8) (yellow).

(d) Binding of $CD_{260\text{-}290}^{2KE}$ mutant to 75%DMPC + 25%DMPG liposomes (n=2 independent experiments). After ultracentrifugation, the fractions from the pellet (P) and the supernatant (S) were analyzed by SDS-PAGE and Coomassie blue staining, similar to Figure 1a. The arrow indicates the $CD_{260\text{-}290}^{2KE}$ protein band.

(e) Superimposed 2D ^{1}H-^{15}N TROSY-HSQC spectra of $CD_{260\text{-}290}^{3RK}$ mutant in solution (red) and in the presence of DMPG/DH^{6}PC bicelles (q=0.8) (purple).

(f) Binding of $CD_{260\text{-}290}^{3RK}$ mutant to 75%DMPC + 25%DMPG liposomes (n=2 independent experiments). After ultracentrifugation, the fractions from the pellet (P) and the supernatant (S) were analyzed by SDS-PAGE and Coomassie blue staining. The arrow indicates the $CD_{260\text{-}290}^{3RK}$ protein band.

Source data are provided as a Source Data file.

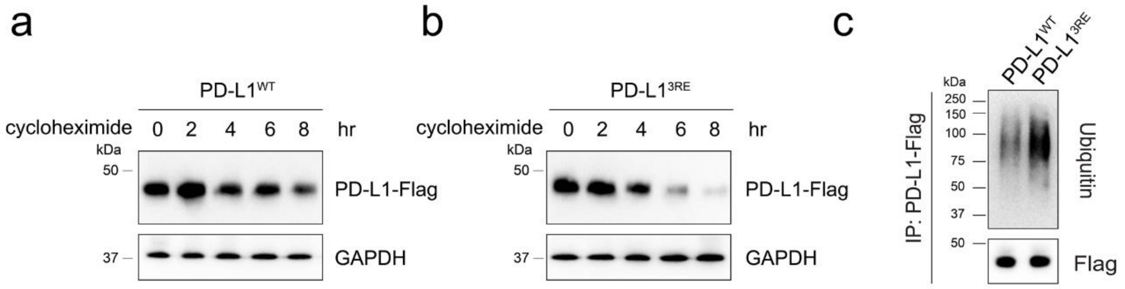

Supplementary Figure 6. The degradation of PD-L1^{WT} or PD-L1^{3RE} mutant in RKO cells was evaluated by CHX-chase assay, related to Figure 4.

(a) RKO cells expressed exogenous PD-L1^{WT} or (b) PD-L1^{3RE} mutant with a Flag tag. The cells were treated with 20 μM cycloheximide (CHX) for 2, 4, 6, or 8 h. The PD-L1 level was analyzed by western blot and identified by Flag antibody to distinguish from the endogenous PD-L1.

(c) Expressing exogenous PD-L1-Flag wildtype or 3RE mutant in RKO cells were lysated and immunoprecipitated by anti-flag antibody. The Ubiquitination were examined by anti-ubiquitin antibody.

All data shown above are representative of two independent experiments.

Source data are provided as a Source Data file.

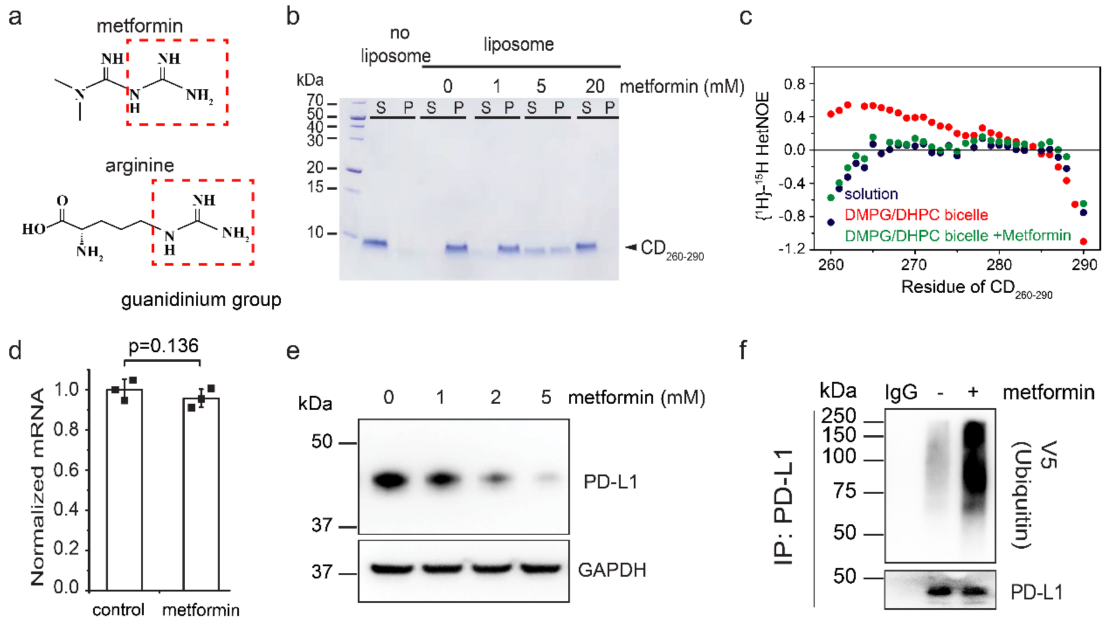

Supplementary Figure 7. Effects of metformin on PD-L1-CD membrane partition and cellular PD-L1 level, related to Figure 5.

(a) Structures of metformin (top) and arginine (bottom). The guanidinium group in both structures are highlighted.

(b) Metformin reduces the liposome binding of $CD_{260\text{-}290}$ (n=2 independent experiments). $CD_{260\text{-}290}$ associated with the liposomes containing 75%DMPC and 25%DMPG in the absence or presence of indicated concentrations of metformin was determined by ultracentrifugation of the samples. The proteins in each fraction were analyzed by SDS-PAGE and Coomassie blue staining similar to Figure 1a.

(c) Heteronuclear $\{^1H\}$-^{15}N NOE (HetNOE) of $CD_{260\text{-}290}$ acquired on the [^{15}N, ^{13}C]-labeled $CD_{260\text{-}290}$ sample in solution (black), DMPG/DH^{6}PC bicelles in the absence (red) or presence (green) of 20 mM metformin using at ^1H frequency of 800 MHz.

(d) mRNA level of PD-L1 in RKO cells treated with 5 mM metformin for 24 h. The mRNA level was measured by RT-PCR. The statistical results from three independent experiment are shown as the mean ± SD.

(e) Cellular PD-L1 level in RKO cells treated with 0, 1, 2, or 5 mM metformin for 24 h determined by western blot (n=3 independent experiments).

(f) PD-L1 ubiquitination in RKO cells treated with 5 mM metformin for 24 h (n=3 independent experiments). PD-L1 were immunoprecipitated (IP) using a PD-L1 antibody and immunoblotted using anti-V5 to detect the V5 tag in ubiquitin.

Source data are provided as a Source Data file.

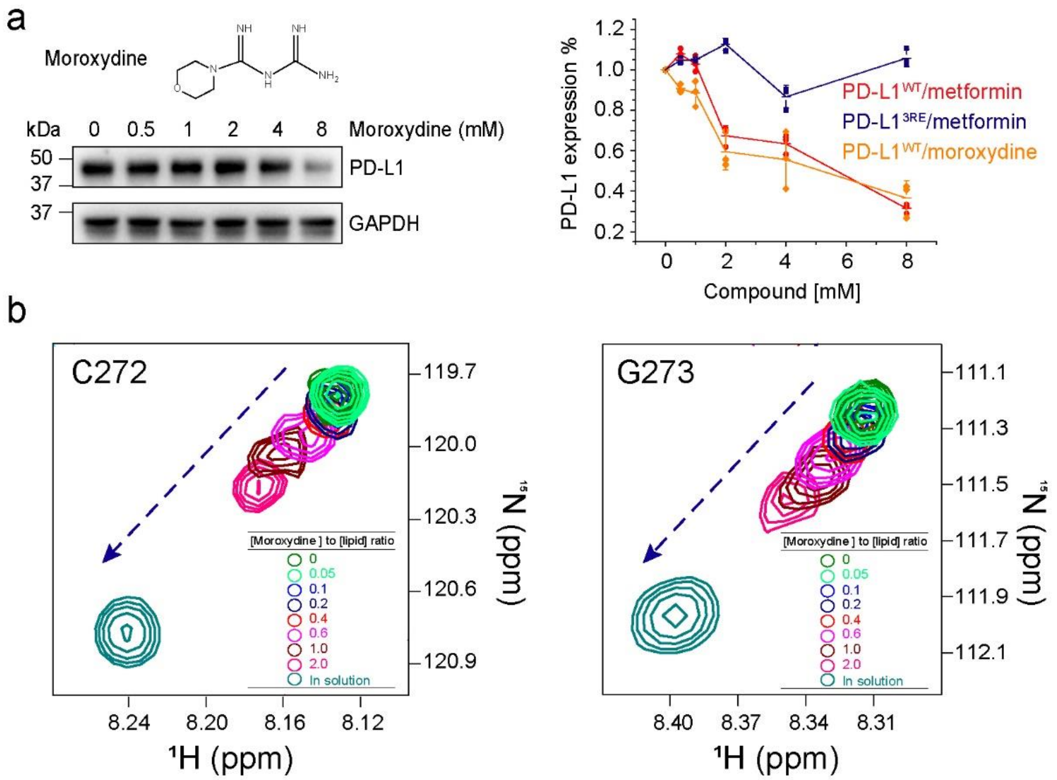

Supplementary Figure 8. Moroxydine shows similar effects to metformin on the membrane association of PD-L1-CD.

(a) Structure of moroxydine with the guanidinium group highlighted, and reduction of PD-L1 level in RKO cells treated with increasing concentrations of moroxydine (0, 0.5, 1, 2, 4, 8 mM) for 24 h detected by western blot (n=3 for each group) (left). The PD-L1 band intensities on the blots were quantified and compared to the metformin effects from Figure 5e (right). Data are represented as mean ± SD.

(b) Dissociation of $CD_{260\text{-}290}$ from DMPG/DH^{6}PC bicelles. Superimposed 2D ^{1}H-^{15}N TROSY-HSQC spectra of $CD_{260\text{-}290}$ for C272 (left) and G273 (right) titrated with different concentrations of moroxydine. The addition of moroxydine shifted the resonances from the position in the DMPG/DH^{6}PC bicelle-bound form (green) to the soluble form (teal).

Source data are provided as a Source Data file.

Supplementary Table 1. Detailed NMR experimental parameters for membrane-bound $CD_{260-290}$.

Experiments	Number of increments (points) and Spectral width (ppm)	#of scans	Delay time (s)	Duration of the experiment	Notes
Backbone assignment					
~1.0 mM (^{15}N,^{13}C, 85% ^{2}H)-labeled PD-L1-CD, DMPG/DH^{6}PC bicelles (q=0.8), 10%D_2O, T=303K, magnetic field: 14.1 T					
2D ^{1}H-^{15}N TROSY HSQC	2048, 14 ($^{1}H^{N}$)<br>256, 21 (^{15}N)	8	1.2	1h 42min	
3D TROSY HNCO	1024, 14 ($^{1}H^{N}$)<br>70, 8 ($^{13}C'$)<br>120, 21 (^{15}N)	8	1.2	6h 58min	NUS-30%
3D TROSY HN(CA)CO	1024, 14 ($^{1}H^{N}$)<br>70, 8 ($^{13}C'$)<br>120, 21 (^{15}N)	16	1.2	1d 8h	NUS-30%
3D TROSY HN(CO)CA	1024, 14($^{1}H^{N}$)<br>100, 28 ($^{13}C^{\alpha}$)<br>120, 21 (^{15}N)	8	1.2	22h 28min	NUS-30%
3D TROSY HNCA	1024, 14($^{1}H^{N}$)<br>100, 28 ($^{13}C^{\alpha}$)<br>120, 21 (^{15}N)	8	1.2	22h 45min	NUS-30%
3D TROSY HNCACB	1024, 14 ($^{1}H^{N}$)<br>80, 60 ($^{13}C^{\alpha/\beta}$)<br>120, 21(^{15}N)	16	1.2	1d 13h	NUS-30%
Protein NOE					
~ 1.0 mM (^{15}N,^{13}C)-labeled PD-L1-CD, D^{54}-DMPG and D^{22}-DH^{6}PC (q=0.8) , 10% D_2O, T=303K, magnetic field: 18.8 T					
2D ^{1}H-^{15}N HSQC	1338, 14 ($^{1}H^{N}$)<br>256, 42 (^{15}N)	8	1.0	37min	
3D ^{15}N and ^{13}C-NOESY-HSQC	1338, 14 ($^{1}H^{N}$)<br>256, 13 (^{1}H)<br>90, 24(^{13}C)<br>90, 59.6(^{15}N)	16	1.16	5d 23h	τ_m = 120 ms
~ 1.0 mM (^{15}N,^{13}C)-labeled PD-L1-CD, D^{54}-DMPG and D^{22}-DH^{6}PC (q=0.8) , 100% D_2O, T=303K, magnetic field: 18.8 T					
3D H(C)CH-TOCSY	1338, 14 ($^{1}H^{N}$)<br>96, 8 (^{1}H)<br>90, 24 (^{13}C)	8	2.3	1d 23h	

3D (H)CCH-TOCSY	1338, 14($^1H^N$)<br>140, 80 (^{13}C)<br>90, 24 (^{13}C)	12	2.3	4d 6h	
3D^{13}C-NOESY-HSQC	1338, 14 ($^1H^N$)<br>256, 13 (^1H)<br>90, 24 (^{13}C)	16	1.16	5d 23h	τ_m = 120 ms
Protein-lipid NOE					
~1.0 mM (^{15}N,^2H)-labeled PD-L1-CD, POPG and D^{22}-DH^{6}PC (q=0.8), 10% D_2O, T=303K, magnetic field: 18.8 T					
2D ^1H-^{15}N TROSY HSQC	1338,14 ($^1H^N$)<br>256,20 (^{15}N)	8	1.0	38min	
3D ^{15}N NOESY-HSQC	1338,14 ($^1H^N$)<br>256,13 (^1H)<br>100,20 (^{15}N)	16	1.5	8d 14h	τ_m = 200 ms

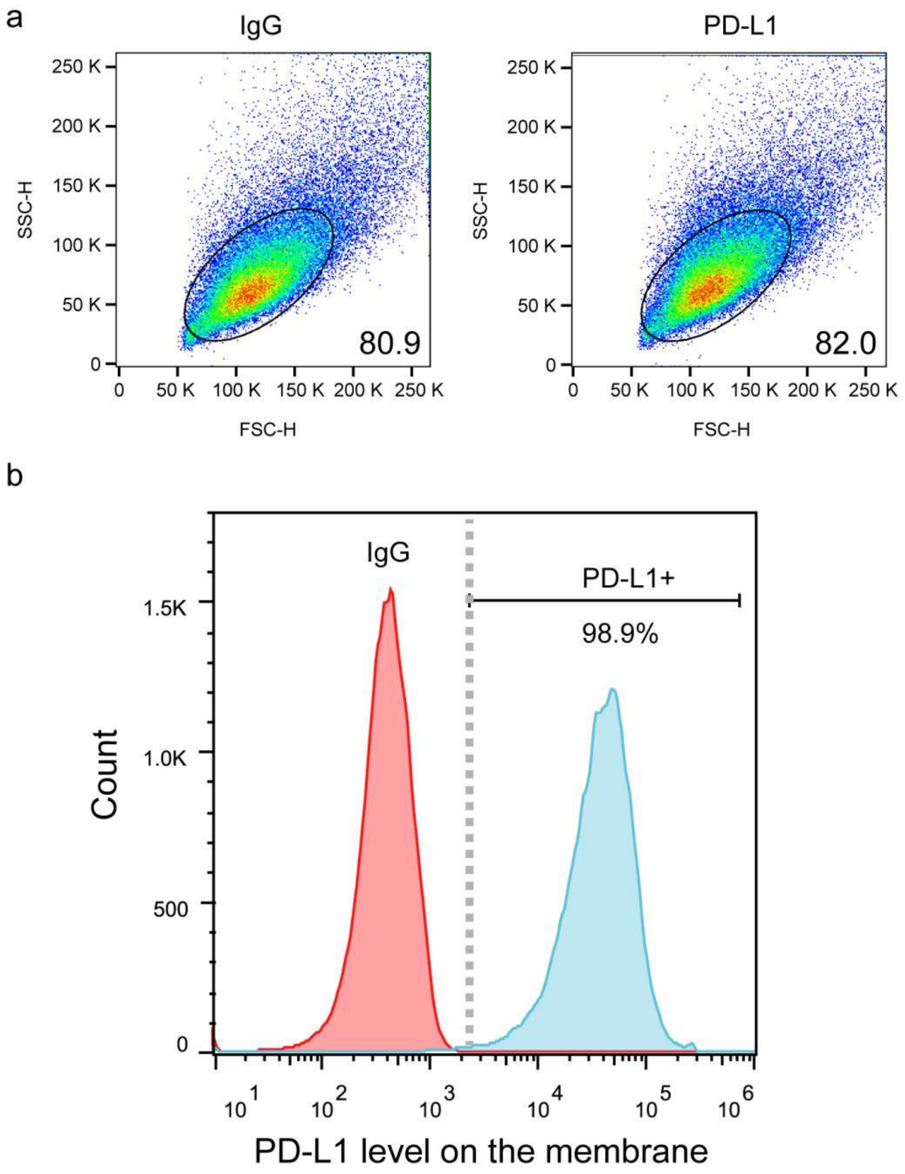

Supplementary Figure 9. Gating strategy for analyzing PD-L1 expression.

(a) The SSC vs FSC 2D-density plot, with gate drawn around the RKO cell population. (b) The me an fluorescence of the FITC-conjugated anti-PD-L1 antibody (blue) was determined and compared with IgG-treated cells as a negative control (red). The boundaries between PD-L1+ and “negative” staining are also indicated in figures.

Supplementary References:

1. Crooks GE, Hon G, Chandonia JM, Brenner SE. WebLogo: a sequence logo generator. *Genome research* 2004, **14**(6)**:** 1188-1190.

2. Kumar S, Stecher G, Tamura K. MEGA7: Molecular Evolutionary Genetics Analysis Version 7.0 for Bigger Datasets. *Molecular biology and evolution* 2016, **33**(7)**:** 1870-1874.
