# Supplementary material for: PD-L1 degradation is regulated by electrostatic membrane association of its cytoplasmic domain
Source: Nat Commun. 2021 Aug 24;12:5106. doi: 10.1038/s41467-021-25416-7 (PMC8384847; doi:10.1038/s41467-021-25416-7)
Supplement: Supplementary file 3 — Reporting Summary [file 41467_2021_25416_MOESM3_ESM.pdf]

nature research

Corresponding author(s): Maorong Wen and Bo OuYang

Last updated by author(s): Aug 5, 2021

Reporting Summary

Nature Research wishes to improve the reproducibility of the work that we publish. This form provides structure for consistency and transparency in reporting. For further information on Nature Research policies, see our Editorial Policies and the Editorial Policy Checklist.

Statistics

For all statistical analyses, confirm that the following items are present in the figure legend, table legend, main text, or Methods section.

n/a	Confirmed	
☐	☒	The exact sample size (n) for each experimental group/condition, given as a discrete number and unit of measurement
☐	☒	A statement on whether measurements were taken from distinct samples or whether the same sample was measured repeatedly
☐	☒	The statistical test(s) used AND whether they are one- or two-sided<br>*Only common tests should be described solely by name; describe more complex techniques in the Methods section.*
☒	☐	A description of all covariates tested
☒	☐	A description of any assumptions or corrections, such as tests of normality and adjustment for multiple comparisons
☐	☒	A full description of the statistical parameters including central tendency (e.g. means) or other basic estimates (e.g. regression coefficient) AND variation (e.g. standard deviation) or associated estimates of uncertainty (e.g. confidence intervals)
☐	☒	For null hypothesis testing, the test statistic (e.g. F, t, r) with confidence intervals, effect sizes, degrees of freedom and P value noted<br>*Give P values as exact values whenever suitable.*
☒	☐	For Bayesian analysis, information on the choice of priors and Markov chain Monte Carlo settings
☒	☐	For hierarchical and complex designs, identification of the appropriate level for tests and full reporting of outcomes
☒	☐	Estimates of effect sizes (e.g. Cohen's d, Pearson's r), indicating how they were calculated

Our web collection on statistics for biologists contains articles on many of the points above.

Software and code

Policy information about availability of computer code

Data collection	NMR data collection: Topspin 3.5, VnmrJ BioPack 4.2<br>Confocal imaging: ZEN (2012, black edition)<br>Flow cytomertry: BD FACSDiva (V8.0)
Data analysis	NMR spectra processing,: NMRpipe 9.7, SMILE (plug-in for NMRPipe package)<br>NMR spectra analysis Sparky 3.115, XEASY 1.2<br>Statistical analysis: Origin 8.0<br>Imaging data analysis: ZEN (2012, black edition), ImageJ (ver. 1.47)<br>Flow cytomertric analysis: FlowJo (VersionX.0.7)

For manuscripts utilizing custom algorithms or software that are central to the research but not yet described in published literature, software must be made available to editors and reviewers. We strongly encourage code deposition in a community repository (e.g. GitHub). See the Nature Research guidelines for submitting code & software for further information.

Data

Policy information about availability of data

All manuscripts must include a data availability statement. This statement should provide the following information, where applicable:
- Accession codes, unique identifiers, or web links for publicly available datasets
- A list of figures that have associated raw data
- A description of any restrictions on data availability

1H, 13C, and 15N chemical shifts have been deposited in the Biological Magnetic Resonance Bank under accession number BMRB 36293 [https://doi.org/10.13018/BMR36293] (Membrane-bound human PD-L1 cytoplasmic domain). All raw and processed data will be made available upon request. Source data are provided with

this paper.

Field-specific reporting

Please select the one below that is the best fit for your research. If you are not sure, read the appropriate sections before making your selection.

☒ Life sciences ☐ Behavioural & social sciences ☐ Ecological, evolutionary & environmental sciences

For a reference copy of the document with all sections, see nature.com/documents/nr-reporting-summary-flat.pdf

Life sciences study design

All studies must disclose on these points even when the disclosure is negative.

Sample size	No statistical methods were used to predetermine sample size. Sample size for each experiment was indicated in legends. Sample size was chosen based on previous experimental experience with similar assays and/or sized generally employed in the field (Wei Yang et al. 2017 (https://doi.org/10.1038/nsmb.3489), Xu C et al. 2008 (10.1016/j.cell.2008.09.044)).
Data exclusions	No data were excluded from the analysis except Western blot. The data when cells were treated for a long time and cell death was detected were excluded from the analysis.
Replication	Number of independent experiments and replicates are stated in the legends.
Randomization	All samples used in this study, including cultured cells, were allocated randomly to each condition.
Blinding	Evaluation for fluorescence intensities from microscopic analysis of splicing reporter assay was conducted in a blind manner. For RT-PCR, western blot, and biochemical assay, cell or compound types were known when prepare the samples or set up the assay.

Reporting for specific materials, systems and methods

We require information from authors about some types of materials, experimental systems and methods used in many studies. Here, indicate whether each material, system or method listed is relevant to your study. If you are not sure if a list item applies to your research, read the appropriate section before selecting a response.

Materials & experimental systems

n/a	Involved in the study	
☐	☒	Antibodies
☐	☒	Eukaryotic cell lines
☒	☐	Palaeontology and archaeology
☒	☐	Animals and other organisms
☒	☐	Human research participants
☒	☐	Clinical data
☒	☐	Dual use research of concern

Methods

n/a	Involved in the study	
☒	☐	ChIP-seq
☐	☒	Flow cytometry
☒	☐	MRI-based neuroimaging

Antibodies

Antibodies used	Anti-human PD-L1 (Abcam, Cat#ab213524), Alexa488-conjugated anti-IgG (Abcam, Cat#ab150077) and Rabbit anti IgG-HRP (Abcam, Cat#ab97051), Anti-V5 (Thermo, Cat#96025), FITC anti PD-L1 (Biolegend, Cat#393606)
Validation	The antibodies were purchased from Abcam, Thermo and Biolegend. All the antibodies used are from commercial sources and have been validated by the vendors. Validation data are available on the manufacturer's website: Anti-human PD-L1 (Abcam, Cat#ab213524, https://www.abcam.cn/pd-l1-antibody-epr19759-ab213524.html), Alexa488-conjugated anti-IgG (Abcam, Cat#ab150077,https://www.abcam.cn/goat-rabbit-igg-hl-alexa-fluor-488-ab150077.html) and Rabbit anti IgG-HRP (Abcam, Cat#ab97051), Anti-V5 (Thermo, Cat#96025, https://www.thermofisher.cn/cn/zh/home/references/protocols/proteins-expression-isolation-and-analysis/antibody-protocol/anti-v5-and-anti-v5-hrp-antibody.html), FITC anti PD-L1 (Biolegend, Cat#393606,https://www.biolegend.com/en-us/products/fitc-anti-human-cd274-b7-h1-pd-l1-antibody-16037?GroupID=BLG9934).

Eukaryotic cell lines

Policy information about cell lines

Cell line source(s)	RKO cell lines were provided by Dr. Jie Xu from Fudan University, HEK293T cell lines were provided by Dr. Liming Sun from CAS Center for Excellence in Molecular Cell Science, all of these cell lines were originally purchased from ATCC. other cell lines used in the paper were from Cell Bank of Chinese Academy of Sciences.

Authentication	None of the cell lines used were authenticated.
Mycoplasma contamination	Cell lines were not tested for mycoplasma contamination.
Commonly misidentified lines (See ICLAC register)	No commonly misidentified cell lines were used.

Flow Cytometry

Plots

Confirm that:

- [x] The axis labels state the marker and fluorochrome used (e.g. CD4-FITC).
- [x] The axis scales are clearly visible. Include numbers along axes only for bottom left plot of group (a 'group' is an analysis of identical markers).
- [x] All plots are contour plots with outliers or pseudocolor plots.
- [x] A numerical value for number of cells or percentage (with statistics) is provided.

Methodology

Sample preparation	Cells were harvested and suspended in 200 μL PBS buffer after washing twice and incubating with anti-human PD-L1 antibody (1:100) at 4 ℃ for 30 min. After washing twice by PBS, the cells were stained by Alexa488-conjugated secondary antibodies (goat-anti-rabbit IgG) were diluted in PBS (1:500) at 4 ℃ for 30 min. After washing twice by PBS, stained cells were analyzed by flow cytometry (BD, LSRFortessa).
Instrument	BD LSRFortessa (BD Biosciences)
Software	Flow cytomertry: BD FACSDiva (V8.0)<br>Flow cytomertric analysis: FlowJo (VersionX.0.7)
Cell population abundance	20000 cells per independent sample
Gating strategy	FSC/SSC gates are exemplified in Supplementary figures. FITC-conjugated corresponding IgG stained cells were used as a negative control. The boundaries between PD-L1+ and "negative" staining are also indicated in Supplementary figures. Then the population was projected to other samples.

- [x] Tick this box to confirm that a figure exemplifying the gating strategy is provided in the Supplementary Information.
